# Identification, Characterization, and Function Analysis of the Cactus Gene from *Litopenaeus vannamei*

**DOI:** 10.1371/journal.pone.0049711

**Published:** 2012-11-21

**Authors:** Chaozheng Li, Yi-Xiao Chen, Shuang Zhang, Ling Lü, Yi-Hong Chen, Jiaoting Chai, Shaoping Weng, Yong-Gui Chen, Jianguo He, Xiaopeng Xu

**Affiliations:** 1 MOE Key Laboratory of Aquatic Product Safety/State Key Laboratory for Biocontrol, School of Life Sciences, Sun Yat-sen University, Guangzhou, P. R. China; 2 School of Marine Sciences, Sun Yat-sen University, Guangzhou, P. R. China; 3 Medical School, Soochow University, Soochow, P. R. China; Ben-Gurion University of the Negev, United States of America

## Abstract

The nuclear factor-kappa B (NF-κB) pathways play important roles in innate immune responses. IκB is the main cytoplasmic inhibitor of NF-κB. In this study, we identified the LvCactus gene from *Litopenaeus vannamei*, which is the first cloned IκB homologue in subphylum Crustacea. LvCactus contains six predicted ankyrin repeats, which show similarities to those of Cactus proteins from insects. LvCactus localizes in cytoplasm and interacts with LvDorsal, an *L. vannamei* homologue to *Drosophila melanogaster* Dorsal belonging to class II NF-κB family, to prevent its nuclear translocation. Contrary to that of LvDorsal, over-expression of LvCactus down-regulates the activities of shrimp antimicrobial peptides promoters, suggesting LvCactus is an inhibitor of LvDorsal. The promoter of LvCactus was predicted to contain five putative NF-κB binding motifs, among which four were proved to be bound by LvDorsal by chromatin immunoprecipitation assays. Dual-luciferase reporter assays also showed that transcription of LvCactus was promoted by LvDorsal but inhibited by LvCactus itself, indicating a feedback regulatory pathway between LvCactus and LvDorsal. Expression of LvCactus was up-regulated after Lipopolysaccharides, poly (I:C), *Vibrio parahaemolyticus*, and *Staphylococcus aureus* injections, suggesting an activation response of LvCactus to bacterial and immune stimulant challenges. Differently, the LvCactus expression levels obviously decreased during white spot syndrome virus (WSSV) infection, indicating the feedback regulatory pathway of LvCactus/LvDorsal could be modified by WSSV.

## Introduction

Pacific white shrimp, *Litopenaeus vannamei*, the main aquacultured shrimp species in the world, belongs to the Penaeidae family of decapod crustaceans [1], [2]. In recent 20 years, a growing number of studies have been focused on breeding, growth, immunity, genetics and evolution of *L. vannamei*, because of its great economic value and important evolutionary status [3]–[8]. Shrimps are susceptible to a wide range of pathogens, including parasites, fungi, bacteria, and viruses, among which bacteria and viruses bring the most challenges to farming industry. With the increase of the stoking density, frequent outbreaks of bacteria and viruses infectious diseases have caused serious economic losses to worldwide *L. vannamei* aquaculture [9]–[11].

In recent year, three major responses against microorganism infection in shrimp have been reported: phagocytosis and encapsulation of invading microorganisms by circulating blood cells; coagulation and phenoloxidase cascades; and the rapid and transient synthesis of antimicrobial peptides [12]–[16]. Research over the last 20 years has shown that innate immunity against bacteria and fungi is governed largely by two nuclear factor-kappa B (NF-κB) signal transduction pathways, Toll and IMD, especially the Toll pathway in shrimp [14], [17]–[21]. Penaeidins are a family of antimicrobial peptides constitutively produced and stored in the hemocytes of shrimp [22]. The upstream promoter regions of these genes contain sequences similar to NF-κB binding motifs [23]. During microbial infections in *L. vannamei*, the Toll/NF-κB pathway is activated and results in the nuclear-translocation and promoter-binding of the Rel transcription factor LvDorsal, a homologue to *Drosophila melanogaster* Dorsal, belonging to class II NF-κB family, followed by promotion of the downstream penaeidins expression [19].

NF-κB is a major transcription factor that can translocate into the nucleus and bind specific promoter motifs to regulate expression of a large number of genes that are involved in many biological processes, such as immune response, apoptosis, cell growth, proliferation, differentiation, and tumor development [24]–[26]. The inhibitor of kappa B (IκB) is a cytoplasmic NF-κB regulator that binds with NF-κB to form a complex and prevents nuclear translocation of NF-κB. NF-κB migrates into the nucleus and regulates biological processes only if IκB is phosphorylated, ubiquitinated and degraded by the proteasome upon stimulation [27]–[30]. In this paper, we identified an IκB homologue gene LvCactus in *L. vannamei* and studied its functions during the immune response. We showed that LvCactus can interact with LvDorsal and prevent its nuclear translocation. Dual-luciferase reporter assays demonstrated that LvCactus can inhibit antimicrobial peptide (AMP) expression, and the expression of LvCactus is promoted by LvDorsal but inhibited by LvCactus itself. Moreover, real-time RT-PCR demonstrated that LvCactus expression responds to Lipopolysaccharides (LPS), *V. parahemolyticus*, *S. aureus*, ploy (I:C) and white spot syndrome virus (WSSV) challenges. As LvCactus is the first cloned IκB homologue gene in subphylum Crustacea, studies on it will help us learn more about the NF-κB pathway in *L. vannamei* and the immune response mechanism of crustaceans.

**Table 1:** Summary of Primers in this study.

Name	Primer sequence (5′–3′)
RACE	
LvCactus 3RACE1	TGGCGTTTTGAATAGCAGCATCAGGT
LvCactus 3RACE2	GATGTTGGAAAGTTACAGCTGCAGTCA
LvCactus 5RACE1	TCGAGGAGGACACAAGGTTTGAACCAG
LvCactus 5RACE2	AGTCTGTAGTAGGGGATGAAACCTTGG
Real-time RT-PCR	
LvEF-1α-F	TATGCTCCTTTTGGACGTTTTGC
LvEF-1α-R	CCTTTTCTGCGGCCTTGGTAG
LvCactus-F	GGAGGCGTGCCAGTGACTATG
LvCactus-R	GAAGTAACGATCTGCATTGAAGGG
Genome walking	
5′GW-LvCactus-1	TCACTAAGGCACACACATGGCCACG
AP1	GTAATACGACTCACTATAGGGC
5′GW-LvCactus-2	CAAGCACCACCATAAACATAACCACTC
AP2	ACTATAGGGCACGCGTGGT
Protein expression	
LvCactus-F	GGGGTACCATCAAAATGTGGCACATTGGCAGTGCCCA
LvCactus-R	TTGGGCCCGAAGTAACGATCTGCATTGAAGG
LvDorsal-F	GGGGTACCATCAAAATGTTTGTTGCCCAGCGTACTTC
LvDorsal-R	TTGGGCCCATATCAGAAAATATCCAAAACTTACCC
GFP-F	GGTTCGAAATCAAAATGGTGAGCAAGGGCGAGGAG
GFP-R	TTGTTTAAACTTACTTGTACAGCTCGTCCATGC
wsv249 promoter-F	CGCAGATCTCTCGCCCACCACCAG
wsv249 promoter-R	GGGGTACCGGCTGCGAGAATGGTTTG
Dual-luciferase reporter assay	
PGL3-LvCactusp-F	GGGGTACCCCATACACAAGAATGCCTCCTGG
PGL3-LvCactusp-R	GAAGATCTCTTGGGGTCAGGGCTTCTGTG
Chromatin immunoprecipitation (for promoter regions relative to the transcription start site (+1))	
LvPen4 (−490 to −49)-F	ACACACAACGCTCATCGGGTC
LvPen4 (−490 to −49)-R	TGTGCAAGCAACTTGGGAGAG
LvCactus-I (−1192 to −964)-F	CCATACACAAGAATGCCTCCTG
LvCactus-I (−1192 to −964)-R	ACAGACTTGTAGGACACGCATACG
LvCactus-II (−1036 to −929)-F	AGCATTTCGACTTGCCCATAC
LvCactus-II (−1036 to −929)-R	AGCTATTGAAGAAATCATTTCCT
LvCactus-III (−951 to −872)-F	AGGAAATGATTTCTTCAATAGCT
LvCactus-III (−951 to −872)-R	ATAAGCTAGCCCTAAATATTTTCCG
LvCactus-IV (−918 to −456)-F	GCGCAATTCACACTAAAGAAAAC
LvCactus-IV (−918 to −456)-R	CCTGTCGCAGTATTGCCAAATG
LvCactus-V (−530 to −356)-F	TTCCTTGTGATAAAACTGCGTAAC
LvCactus-V (−530 to −356)-R	CCAACTTTCTCATCATTCCAAGG
LvCactus-VI (−431 to −186)-F	TGCCACCTCTCACCGGAATTG
LvCactus-VI (−431 to −186)-R	TTGATTGTACTGCTGGTAGTAAATG
LvCactus-VII (−237 to −64)-F	GGAATGTCGTCACAATTAGCTGC
LvCactus-VII (−237 to −64)-R	CGTCACCACTCAACTATGTGCG
LvCactus-VIII (−120 to +22)-F	TTCCAAATGTGAAACAAATACAGTG
LvCactus-VIII (−120 to +22)-R	GTGAAGTCTACTGAGCGTGTGTGG

## Materials and Methods

### Cloning of LvCactus cDNA

Based on data from the *L. vannamei* transcriptome analyzed by our lab [31], a sequence that was predicted to encode a Cactus homologous protein was obtained and used to design specific primers to clone the LvCactus gene (Table 1). Briefly, Total RNA was extracted from *L. vannamei* hemocytes with Trizol (Invitrogen, USA) and treated with RNase-free DNase (Promega, USA). Rapid amplification cDNA ends (RACE) were then performed using the SMARTer^TM^ RACE cDNA Amplification kit (Clontech, Japan) according to the manufacturer's protocol. 5′-Rapid amplification of cDNA ends (RACE)-PCR amplification was performed with Universal Primer A Mix (UPM) and LvCactus specific reverse primer 5RACE1. Nested PCR was subsequently performed with Nested Universal Primer A (NUP) and LvCactus 5RACE2 using the first-round PCR product as template. 3′-RACE-PCR was performed using UPM together with an LvCactus-specific forward primer 3RACE1, and the nested PCR was subsequently performed with NUP and LvCactus 3RACE2. The second PCR products were cloned into pMD-20T vector (TaKaRa, Japan) and 12 positive clones were selected and sequenced (ABI PRISM, Applied Biosystems, USA).

**Figure 1:**
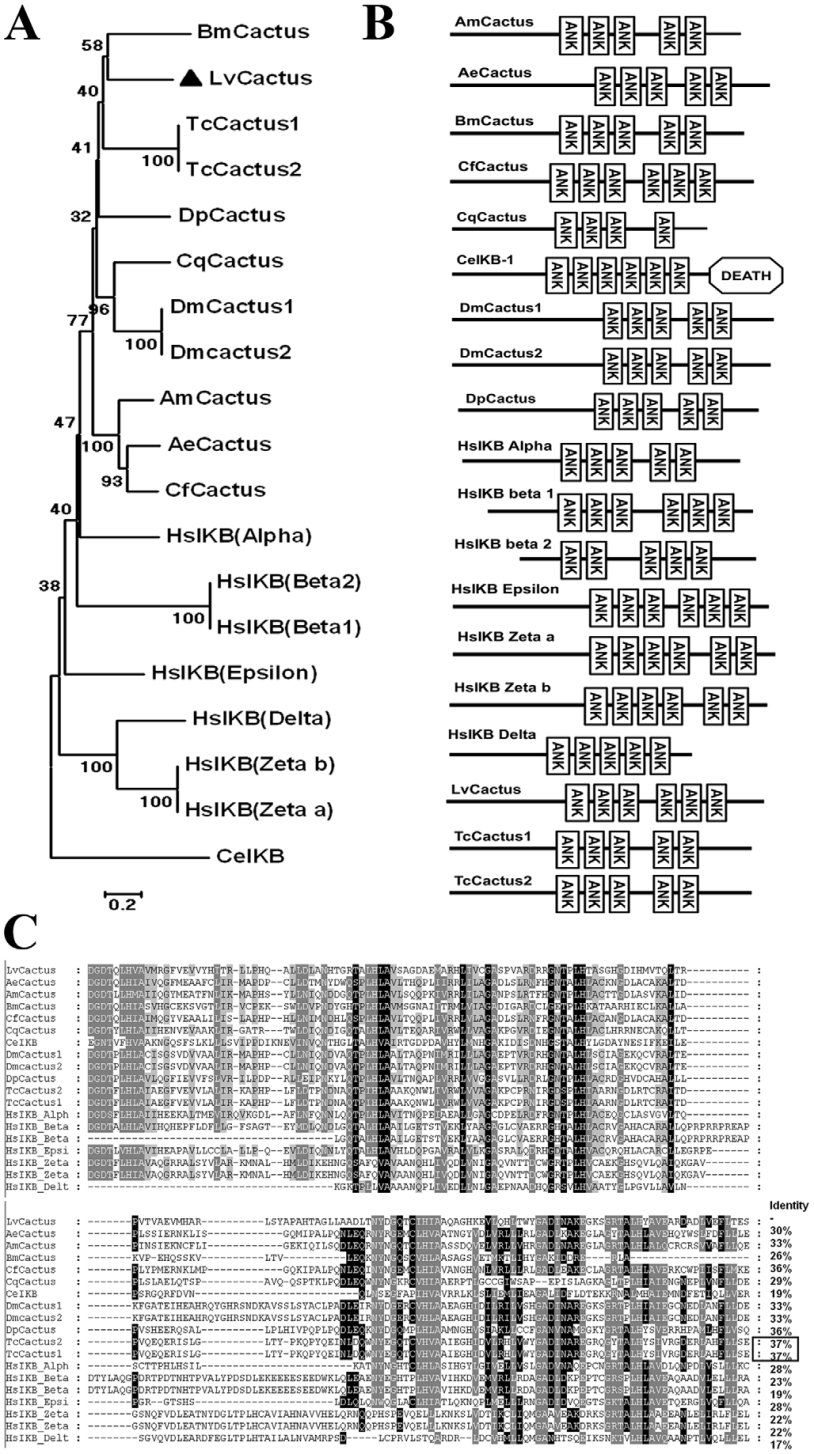
Phylogenetic tree construction and multiple sequence alignment of Cactus proteins from various species. (A) Neighbor-joining phylogenetic tree analysis of the full-length amino acid sequences of Cactus proteins (LvCactus was marked with solid triangle) using MEGA 5.0 software; (B) Schematic representation and (C) Multiple sequence alignment (using clustal X v2.0 method) of the ankyrin repeat domains of Cactus proteins with the identical amino acid residues shaded in black and the similar residues in gray. Proteins analyzed list below: LvCactus, *Litopenaeus vannamei* Cactus (Accession No. JX014314); AmCactus, *Apis mellifera* Cactus 1 (Accession No. NP_001157184.1); AeCactus, *Acromyrmex echinatior* Cactus (Accession No. EGI65248.1); BmCactus, *Bombyx mori* Cactus (Accession No. NP_001166191.1); CfCactus, *Camponotus floridanus* Cactus (Accession No. EFN66754.1); CeIκB-1, *Caenorhabditis elegans* IκB-1 (Accession No. NP_492575.1); DmCactus1, *Drosophila melanogaster* Cactus isoform A (Accession No. AAN10936.1); DmCactus2, *Drosophila melanogaster* Cactus isoform B (Accession No. NP_476942.1); DpCactus, *Daphnia pulex* Cactus (Accession No. EFX89207.1); HsIκB Alpha, *Homo sapiens* IκB Alpha (Accession No. NP_065390.1); HsIκB beta1, *Homo sapiens* IκB beta isoform 1 (Accession No. NP_002494.2); HsIκB beta2, *Homo sapiens* IκB beta isoform 2 (Accession No. NP_001230045.1); HsIκB Epsilon, *Homo sapiens* IκB Epsilon (Accession No. NP_004547.2); HsIκB Zeta a, *Homo sapiens* IκB Zeta isoform a (Accession No. NP_113607.1); HsIκB Zeta b, *Homo sapiens* IκB Zeta isoform b (Accession No. NP_001005474.1); HsIκB Delta, *Homo sapiens* IκB Delta (Accession No. NP_640332.1); TcCactus1, *Tribolium castaneum* Cactus isoform 1 (Accession No. NP_001157183.1) and TcCactus2, *Tribolium castaneum* Cactus isoform 2 (Accession No. NP_001157182.1).

### Genome walking

The *L. vannamei* genome DNA was prepared according to the protocol as previously described [32]. Genome walking libraries were constructed by GenomeWalker™ Universal Kit (Clontech, Japan) according to the user manual. The primer pairs AP1/5′GW-LvCactus-1 and AP2/5′GW-LvCactus-2 were used to perform the first and second rounds of genome walking PCR amplification, respectively.

**Figure 2:**
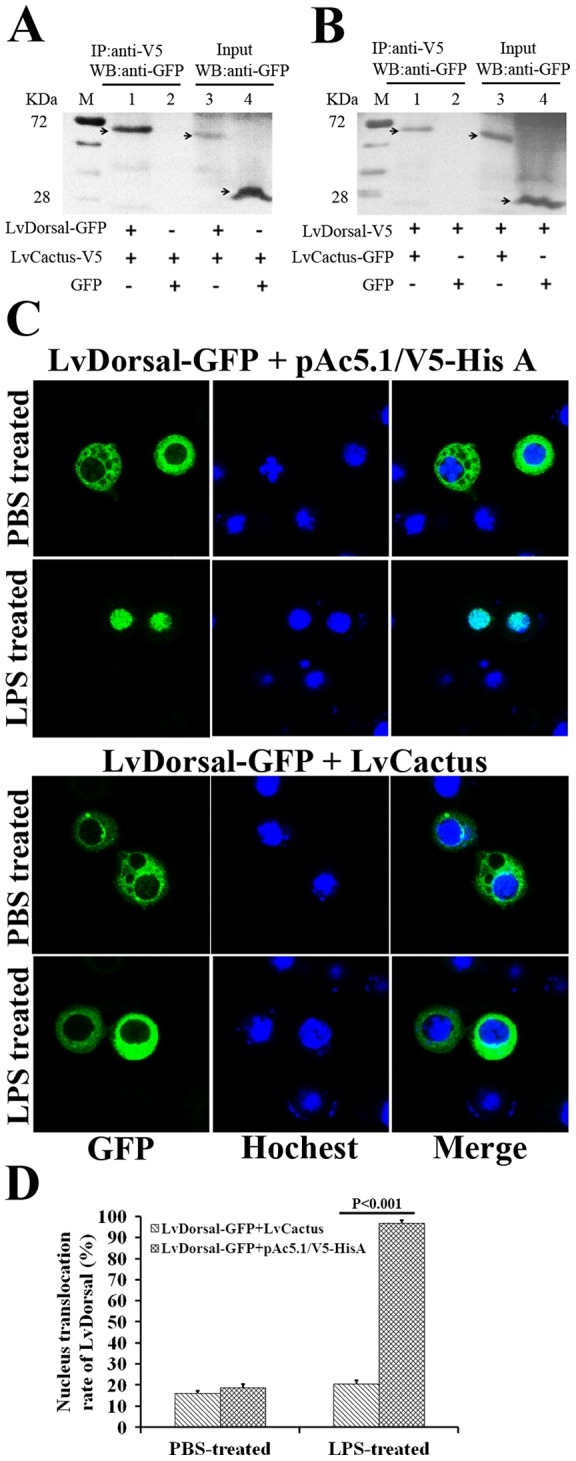
Interaction between LvCactus and LvDorsal. (A). Co-immunoprecipitation assays showed that the GFP-tagged LvDorsal but not the control GFP protein can be co-precipitated by V5-tagged LvCactus. (B). Reciprocal co-immunoprecipitation showed that GFP-tagged LvCactus but not GFP can be co-precipitated with V5-tagged LvDorsal. Immunoprecipitation (IP) and western-blotting were performed using anti-V5 and anti-GFP antibodies, respectively. Input: western-blotting analysis of the input cell lysates (3%) before immunoprecipitation. (C) LvCactus prevents cytoplasm-to-nucleus translocation of LvDorsal in response to LPS stimulation. S2 cells were transfected with pAc5.1-LvDorsal-GFP together with pAc5.1-LvCactus-V5 or empty plasmid pAc5.1/V5-His A (as control), and 24 h later treated with 1 μg/mL LPS or PBS (as control) for 6 h. The nuclei were stained with Hochest 33258 (blue). (D) The LvDorsal nucleus-translocation rate, determined by calculating the percentage of LvDorsal nucleus-translocation cells in all LvDorsal-expressing cells in three randomly selected visual fields, each containing at least 15 LvDorsal-expressing cells for statistical requirements. The bars indicate the mean ± SD of the data (n = 3).

### Bioinformatics analysis

Protein sequences of Cactus homologues from other species were retrieved from the National Center for Biotechnology Information (NCBI, http://www.ncbi.nlm.nih.gov/) databases using the BLAST program (basic local alignment search tool). Sequence alignments between LvCactus and Cactus homologues from other species were analyzed using clustal X v2.0 program [33]. Phylogenetic trees were constructed based on the deduced amino acid sequences using MEGA 5.0 software, applying the amino acid substitution type and poisson model and bootstrapping procedure with a minimum of 1000 bootstraps [34]. Protein domains were predicted using the SMART program (http://smart.embl-heidelberg.de/) and the proline-, glutamic acid-, serine-, and threonine-rich (PEST) sequence was identified by ePESTfind (http://emboss.bioinformatics.nl/cgi-bin/emboss/epestfind). Transcription factor binding sites were identified using the PROMO program with default parameter setting [35].

**Figure 3:**
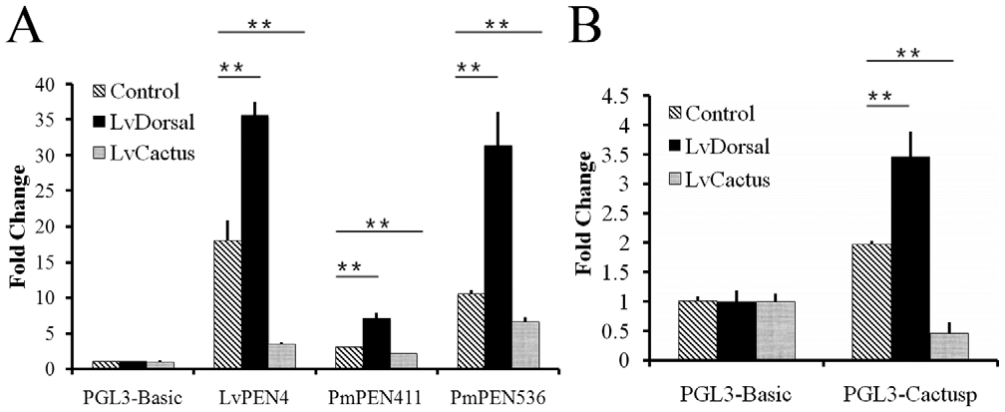
Dual luciferase reporter assays on Drosophila S2 cells. (A) Effects of LvCactus and LvDorsal on promoters of three shrimp antimicrobial peptide (AMP) genes, LvPEN4, PmPEN411 and PmPEN536. LvDorsal enhances the activities of all the three AMPs promoters, whereas LvCactus suppresses them. (B) Effects of LvCactus and LvDorsal on the promoter activity of LvCactus. The LvCactusp promoter was activated by LvDorsal over-expression but inhibited by LvCactus over-expression. The bars indicate the mean ± SD of the luciferase activities (n = 3). The statistical significance was calculated by the Student's t-test (*p<0.05, **p<0.01).

### Plasmid constructions

The GFP coding sequence was cloned into a *Drosophila* expression vector pAc5.1/V5-His A (Invitrogen) at BstBI/PmeI sites to replace the V5-His tag, generating a pAc5.1-GFP vector for GFP-tagged expression. The open reading frames (ORFs) of LvCactus and LvDorsal were cloned into pAc5.1/V5-His A and pAc5.1-GFP vectors at the KpnI/ApaI and KpnI/SacII sites to generate pAc5.1-LvCactus-V5/pAc5.1-LvDorsal-V5 and pAc5.1-LvCactus-GFP/pAc5.1-LvDorsal-GFP for expressing V5- and GFP-tagged proteins, respectively. The p249-LvDorsal-V5 plasmid was derived from pAc5.1-LvDorsal-V5 by replacing the Ac5 promoter with the 331-bp promoter of wsv249 gene from WSSV [36]. All the expression vectors were inserted with a *Drosophila* Kozak translation initiation sequence (ATCAAA) and an ATG initiation codon for proper initiation of translation [37]. The PGL3-LvCactusp was obtained by cloning the promoter sequence of LvCactus into PGL3-Basic vector (Promega) at KpnI/BglII sites. Three vectors of PGL3-AMPs containing promoters of PEN411 and PEN536 from *Penaeus monodon* (PmPEN411 and PmPEN536) and PEN4 from *L. vannamei* (LvPEN4), respectively, were constructed according to previous reports [23], [38].

**Figure 4:**
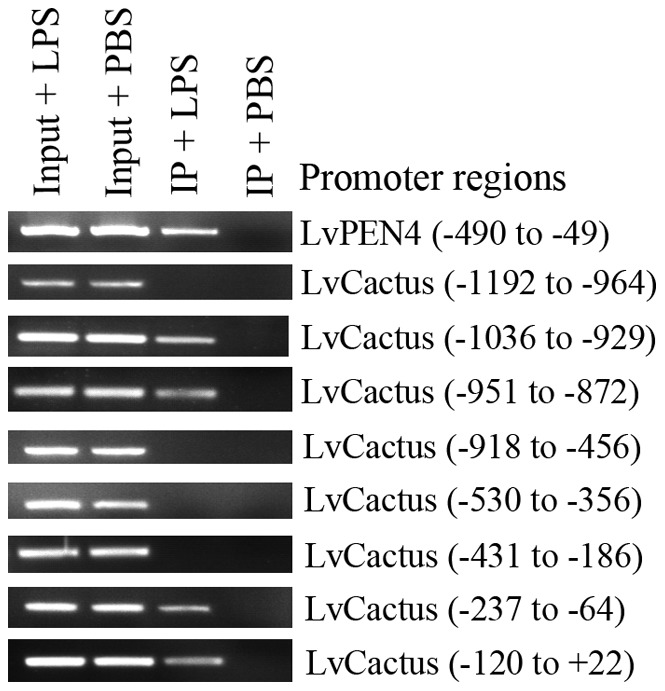
Chromatin Immunoprecipitation assays performed using V5-tagged LvDorsal-expressing primary hemocytes with or without LPS stimulation. The precipitated DNA was subjected to PCR analysis. The promoter region of LvPEN4 and eight fragments of the LvCactus promoter were detected. The positions of the fragments were orientated by the transcription start site (+1). Input: analysis by PCR of the input chromatin (0.2%) before immunoprecipitation.

### Confocal laser scanning microscopy

*Drosophila* Schneider 2 (S2) cells were seeded onto poly-L-lysine-treated cover slips in 6-well plates. To detect subcellular localization of LvCactus, cells were transfected with GFP-fused LvCactus using Effectene Transfection Reagent (Qiagen, Germany). At 48 h post-transfection, the S2 cells were washed three times with PBS, fixed by Immunol Staining Fix Solution (Beyotime, China), and stained with 2 ug/ml Hochest 33258 (Sigma, USA). Fluorescence was visualized and captured with confocal laser scanning microscope (Leica TCS-SP5, Germany) and analyzed using Leica LAS AF Lite software. To analyze the cytoplasm-to-nucleus translocation of LvDorsal, pAc5.1-LvDorsal-GFP was co-transfected with pAc5.1-LvCactus-V5 and pAc5.1/V5-His (as control) into S2 cells. At 24 h post transfection, cell cultures were added with LPS from Escherichia coli 0111:B4 (Sigma) to a final concentration of 1 μg/1mL, or PBS as control. After 6 h, cells were observed with confocal laser scanning microscope. The number of LvDorsal nucleus-translocation cells was measured in three randomly selected visual fields, each containing at least 15 LvDorsal-expressing cells for statistical requirements. The LvDorsal nucleus-translocation rate was determined by calculating the percentage of LvDorsal nucleus-translocation cells in all LvDorsal-expressing cells.

**Figure 5:**
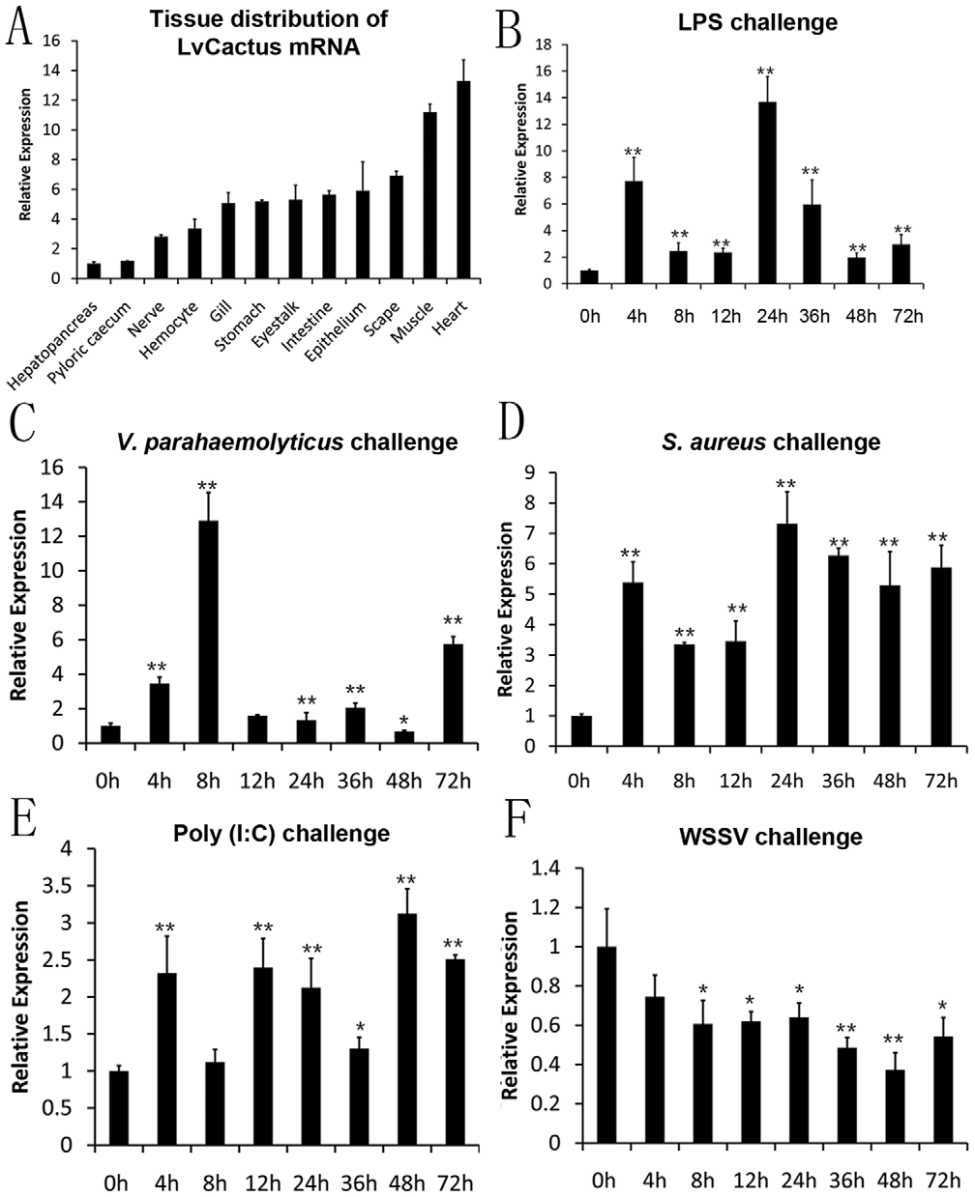
Tissue distribution of LvCactus mRNA in healthy *L. vannamei* and its expression profiles in hemocytes from pathogens or stimulants challenged *L. vannamei*. Real-time RT-PCR was performed in triplicate for each sample. Expression values were normalized to those of LvEF-1α using the Livak (2^−△△CT^) method and the data were provided as the mean fold changes (means ± SE, n = 3) relative to the control group. (A) Transcription levels of LvCactus in different tissues of healthy *L. vannamei*. Expression level in the hepatopancreas was used as control and set to 1.0. (B–F) Expression profiles of LvCactus in hemocytes from LPS, *V. parahemolyticus*, *S. aureus*, poly (I:C), and WSSV challenged shrimps. Expression level at 0 h post injection of each group was set as 1.0.

### Co-immunoprecipitation and Western blot assays

For co-immunoprecipitation, pAc5.1-Lvcactus-V5 was co-transfected with pAc5.1-LvDorsal-GFP and pAc5.1-GFP (as a control) into S2 cells. For reciprocal coimmunoprecipitation, pAc5.1-LvDorsal-V5 was co-transfected with pAc5.1-LvCactus-GFP and pAc5.1-GFP. After 72 h, cells were harvested and lysed in NP-40 lysis buffer with a protease inhibitor cocktail (Sigma). Both co-immunoprecipitation and reciprocal co-immunoprecipitation were performed using anti-V5 agarose affinity gel (Sigma). Western blotting was performed with rabbit anti-GFP antibody (Sigma) and alkaline phosphatase-conjugated goat anti-rabbit secondary antibodies (Sigma). A standardized aliquot (3%) of each total input cell lysates was also examined as control.

**Figure 6:**
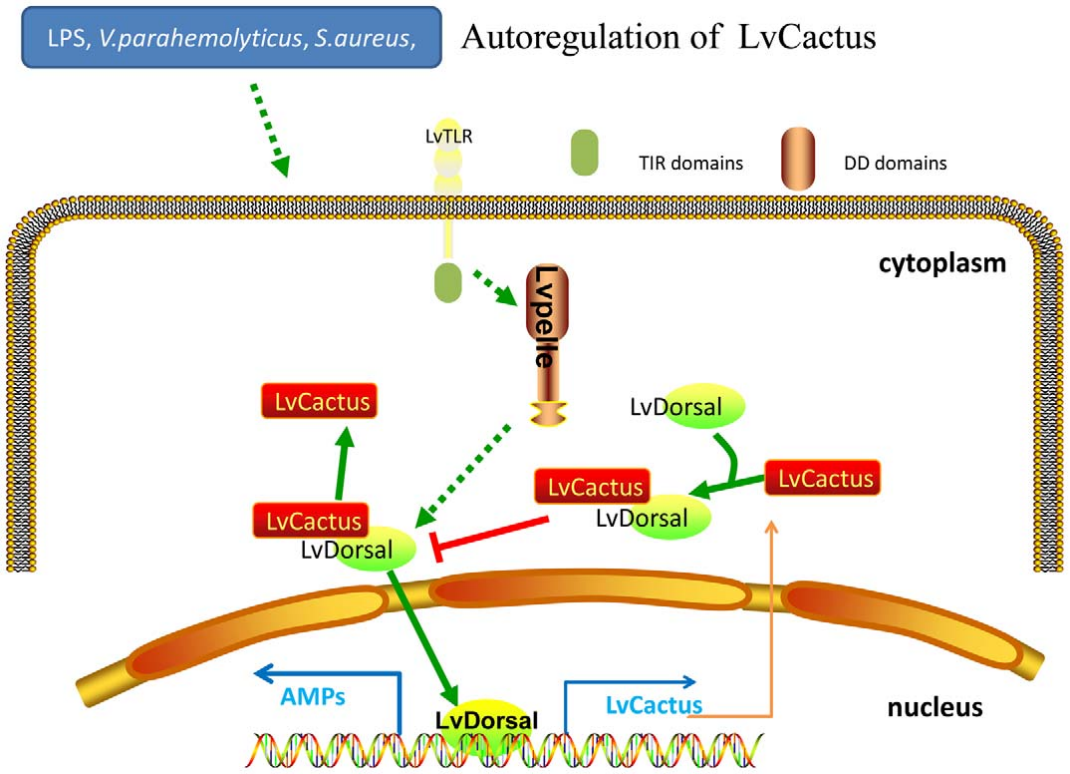
Schematic diagram for the LvCactus/LvDorsal feedback regulatory pathway. For details, see ‘Discussion’.

### Dual-luciferase reporter assays

S2 cells were cultured at 28°C in *Drosophila* SDM (Serum-Free Medium; Invitrogen) supplemented with 10% fetal bovine serum (Invitrogen). For DNA transfection, Cell plating and transfection are performed on the same day, and plasmids were transfected using the Effectene Transfection Reagent (Qiagen) according to the manufacturer's protocol. For dual-luciferase reporter assays, S2 cells in each well of a 96-well plate (TPP, Switzerland) were transfected with 0.05 ug reporter gene plasmids, 0.005 ug pRL-TK renilla luciferase plasmid (Promega), and 0.05 ug expression plasmids or empty pAc5.1/V5-His A plasmid (as control). The pRL-TK renilla luciferase plasmid was used here as an internal control. At 48 hour post transfection, Dual-Luciferase Reporter Assays were performed to measure the firefly and renilla luciferase activities according to the manufacturer's instructions. Each experiment was done at least three times.

### Chromatin Immunoprecipitation (ChIP) Assay

Hemocytes from healthy *L. vannamei* shrimps were collected, suspended in sera-free Leibovitz-15 (L-15, Sigma) growth medium with a density of 1×10^7^/ml, electrotransfected with p249-LvDorsal-V5 by a single pulse of 250V and 15msec on ECM-830 electroporator (BTX Harvard Apparatus, USA). Cells were then cultured in 25 cm^2^ bottles as previously described [15]. The expression of V5-tagged LvDorsal protein was verified using Western-blot analysis. At 72 h post transfection, cells were treated with LPS (final concentration of 1 μg/mL) or PBS (as control), respectively. 12 h later, cells were cross-linked with 1% formaldehyde at room temperature for 10 min and terminated with Glycine Solution (0.025 M Tris, 0.192 M glycine, pH 8.0). Cells were then washed with ice-cold PBS three times and collected into 0.3 ml of lysis buffer (1% SDS, 10 mM EDTA, 50 mM Tris-HCl, pH 8.0, 1× protease inhibitor cocktail (Sigma) and sonicated three times for 10 s each at the maximum setting (Scientz-IID, LifeScientz, China) followed by centrifugation for 10 min at 12000 g. Supernatants were collected and diluted in buffer (1% Triton X-100, 2 mM EDTA, 150 mM NaCl, 20 mM Tris-HCl, pH 8.0) followed by immunoclearing with 2 mg sheared salmon sperm DNA, 20 μl mouse preimmune serum and 60 μl protein A+G agarose (50% slurry in 10 mM Tris-HCl, pH 8.0, 1 mM EDTA) for 1 h at 4°C. Immunoprecipitation was performed for 4 h at 4°C with mouse anti-V5 antibodies (Sigma). After immunoprecipitation, 60 μl protein A+G agarose and 2 μg of salmon sperm DNA were added and the incubation was continued for another 1 h. Precipitates were washed sequentially for 5 min each in Low Salt Immune Complex Wash Buffer (0.1% SDS, 1% Triton X-100, 2 mM EDTA, 20 mM Tris-HCl, pH 8.0, 150 mM NaCl), High Salt Immune Complex Wash Buffer (0.1% SDS, 1% Triton X-100, 2 mM EDTA, 20 mM Tris-HCl, pH 8.0, 500 mM NaCl), and LiCl Immune Complex Wash Buffer (0.25 M LiCl, 1% NP-40, 1% deoxycholate, 1 mM EDTA, 10 mM Tris-HCl, pH 8.0). Precipitates were then washed three times with TE buffer and extracted three times with freshly prepared 1% SDS, 0.1 M NaHCO_3_. Eluates were pooled and heated at 65^°C^ for at 4 h to reverse the formaldehyde cross-linking. DNA fragments were purified with a QIAquick Spin Kit (Qiagen) and subjected to PCR with 21–25 cycles of amplification. Primers were designed to amplify the promoter of LvPEN4 and eight regions of LvDorsal promoter (Table 1). A standardized aliquot (0.2%) of each total input chromatin was also examined as control. The PCR products were analyzed using agarose gel electrophoresis, and subcloned into the PMD-20T vector for Sanger sequencing to confirm the results.

### Immune challenge and real-time RT-PCR analysis

Healthy *L. vannamei* (average 5 g) were obtained from Hengxing shrimp farm in Zhanjiang, China. The hepatopancreas, pyloric caecum, nerve, hemocyte, gill, stomach, eyestalk, intestine, epidermis, scape, muscle and heart tissues from 15 *L. vannamei* were sampled and pooled for tissue expression analysis. For challenge experiments, *L. vannamei* were cultured in freshwater tanks at room temperature (27°C) and divided into 5 experimental groups, in which *L. vannamei* was injected at the second abdominal segment with 2 ug/μl poly (I:C), 2 ug/μl LPS, 10^6^ particles of *Vibrio parahaemolyticus*, 10^6^ particles of *Staphylococcus aureus*, and 10^6^ copies newly extracted WSSV particles in 50 μl DEPC-treated water prepared PBS solution (pH 7.4), respectively [12], as well as a control group injected with 50 μl PBS. Hemocytes of challenged shrimps were sampled at 0, 4, 8, 12, 24, 36, 48, 72 h post injection (hpi), and each time point sample was collected and pooled from 15 shrimps. Total RNA was then isolated with the TRIzol reagent and subsequently reverse transcribed to cDNA using PrimeScript RT Reagent Kit (TaKaRa) according to the manufacturer's instructions. Reactions were performed in the LightCycle 480 System (Roche, Germany) according to the manufacturer's protocol. Real-time RT-PCR assays were performed at a volume of 10 µl comprised of 1 µl of 1∶10 cDNA diluted with ddH_2_O, 5 µl of 2× SYBRGreen Master Mix (Takara, Japan), and 250 nM of each primer. The cycling parameters were 95°C for 2 min to activate the polymerase, followed by 40 cycles of 95°C for 15 s, 62°C for 1 min, and 70°C for 1 s. Cycling ended at 95°C with 5°C/s calefactive velocity to create the melting curve. Fluorescence measurements were taken at 70°C for 1 s during each cycle. Expression levels of LvCactus were calculated using the Livak (2^−△△CT^) method after normalization to EF-1α (GenBank accession no. GU136229). Primer sequences are listed in Table 1.

## Results

### cDNA cloning and sequence analysis of LvCactus

The LvCactus transcript is 2809 bp long, with a 5′-untranslated region(UTR) of 247 bp, a 3′-untranslated region of 1200 bp, and an open reading frame (ORF) of 1362 bp encoding a protein of 453 amino acids with a calculated molecular weight of 48.4 kDa (Genbank Accession No. JX014314). Architecture analysis shows that the LvCactus protein sequence contains an IκB degradation motif (DS_57_GFLS_61_) encompassed by two signal recognition-related serine residues (Ser_57_ and Ser_61_) in the N-terminal region, a PEST sequence in the 121–145 region, and six ankyrin repeats (ANKs) distributed in the 175–398 region (Fig. S1). By the Genome Walking method, we obtained the promoter sequence of LvCactus with 1192 bp long including five putative NF-κB binding sites at positions −961 to −952, −928 to −919, −454 to −444, −142 to −133, and −12 to −1 relative to the transcription start site (+1), which suggest a possible regulation of LvCactus expression by the NF-κB pathway.

### Phylogenetic analysis

The full lengths of the LvCactus protein and its homologues in other species were subjected to phylogenetic analysis by the neighbor-joining (NJ) method using MEGA5.0 software [34]. According to the NJ phylogenetic tree (Fig. 1A), the Arthropoda Cactus proteins used in this study were clustered to a subtree followed by the branches of human IκB proteins and *Caenorhabditis elegans* Cactus proteins. LvCactus was mostly clustered with the insect protein *Bombyx mori* Cactus (BmCactus) and then sub-clustered with *Tribolium castaneum* Cactus isoform 1 and 2 (TcCactus1/2). Multiple sequence alignment showed that the ANK domains of LvCactus are similar to those of TcCactus1/2 (both 37% identity), *Daphnia pulex* Cactus (DpCactus) (36% identity), *Camponotus floridanus* Cactus (CfCactus), and *D. melanogaster* Cactus isoform 1 and 2 (DmCactus1/2) (both 33% identity) (Fig. 1B and C).

### LvCactus is localized in the cytoplasm

To identify the cellular location of LvCactus protein, S2 cells were transfected with plasmid encoding GFP-tagged LvCactus and then stained with Hoechst for nuclear counterstaining followed by observation under confocal laser scanning microscope. The fluorescent signals of the LvCactus–GFP fusion proteins are dispersedly present in the cells and surround the nuclear areas, suggesting LvCactus is a cytoplasm-localized protein (Fig. S2).

### Interaction between LvCactus and LvDorsal

As an IκB homologue, Cactus can bind with the NF-κB homologue Dorsal to form a complex in *D. melanogaster* [39]. To investigate the potential interaction between LvCactus and LvDorsal, coimmunoprecipitation and reciprocal coimmunoprecipitation assays were performed with S2 cells expressing V5-tagged LvCactus/GFP-tagged LvDorsal and GFP-tagged LvCactus/V5-tagged LvDorsal, respectively (Fig. 2A and B). Immunoprecipitation assays showed that the GFP-tagged LvDorsal but not the control GFP protein exhibits affinity to LvCactus and can be co-precipitated by the V5-tagged LvCactus. Reciprocal coimmunoprecipitation also showed that the GFP-tagged LvCactus but not GFP can be co-precipitated with V5-tagged LvDorsal, which confirmed the interaction between LvCactus and LvDorsal.

To determine whether LvCactus can prevent nuclear translocation of LvDorsal, the subcellular localization change of LvDorsal-GFP fusion protein in response to LPS stimulation was detected in S2 cells with or without LvCactus over-expression (Fig. 2C). After mock-treated with PBS, LvDorsal-GFP was observed in the cytoplasm of most of the LvCactus non- and over-expressing cells, and only a small part of the cells (18.7% and 16.1%, respectively) exhibited LvDorsal-nucleus-translocation. After treated with LPS, LvDorsal-nucleus-translocation was observed in 96.9% and 20.5% of the LvCactus non- and over-expressing cells, respectively, with a significant difference between the two groups (P<0.001), suggesting a 78.8% inhibition rate of LvDorsal translocation by LvCactus over-expression (Fig. 2D).

### AMP genes were down-regulated by LvCactus over-expression in S2 cells

As a member of class II NF-κB family, LvDorsal can bind to NF-κB binding DNA motifs and promote the expression of *D. melanogaster* and *L. vannamei* AMP genes [19]. In this study, dual-luciferase reporter assays further confirmed that LvDorsal can up-regulate expressions of AMP genes LvPEN4, PmPEN411 and PmPEN536 with 1.97-, 2.32-, and 2.98-fold increase, respectively (Fig. 3A). To determine whether LvCactus could be an IκB-like inhibitor of LvDorsal, dual-luciferase reporter assays were performed to measure the effect of LvCactus over-expression on transcription of shrimp AMPs. The results showed that transient expression of LvCactus could down-regulate pGL3-LvPEN4 transcription by 5.17-fold, pGL3-PmPEN411 by 1.38-fold, and pGL3-PmPEN536 by 1.57-fold (Fig. 3A), suggesting that LvCactus can suppress AMP responses.

### LvCactus expression was autoregulated

The 1192 bp promoter region of LvCactus, containing putative NF-κB binding DNA motifs, was cloned into PGL3-Basic vector to generate pGL3-LvCactusp and a luciferase reporter assay was performed. The results showed that over-expression of LvDorsal could increase pGL3-LvCactusp expression by 1.75-fold, while over-expression of LvCactus could decrease pGL3-LvCactusp expression by 4.28-fold. In consideration of the interaction between LvCactus and LvDorsal, we concluded that LvCactus could regulate expression of itself through binding and inhibiting LvDorsal (Fig. 3B).

### LvCactus promoter can be bound by LvDorsal

ChIP assays were performed using V5-tagged LvDorsal-expressing primary hemocytes with or without LPS stimulation and the precipitates were examined by PCR (Fig. 4). As a positive control, the promoter region of LvPEN4 gene can be co-precipitated by the V5-tagged LvDorsal in LPS-treated cells, but not in PBS mock-treated cells, indicating that in response to LPS stimulation LvDorsal can translocate into the nucleus and bind with NF-κB binding DNA motifs in AMP gene promoter. To verify the five putative NF-κB binding motifs, LvCactus promoter was divided into eight regions, each with overlapping ends with its contexts, and detected by PCR, respectively. The results showed that four regions locating in −1036 to −929, −951 to −872, −237 to −64, and −120 to +22 relative to the transcription start site (+1) of LvCactus were co-precipitated by LvDorsal in response to LPS stimulation, confirming that LvDorsal can bind with LvCactus promoter to regulate its transcriptional activity. These four regions cover four putative NF-κB binding sites at positions −961 to −952, −928 to −919, −142 to −133, and −12 to −1, suggesting these sites could be bound by LvDorsal.

### Tissue distribution of LvCactus mRNA

The LvCactus mRNA could be detected in all the tissues examined. The relative expression levels of LvCactus in other tissues were normalized to that in hepatopancreas, which was set as 1.0. The results showed that expression of LvCactus is low in hepatopancreas and pyloric caecum, moderate in most tested tissues including the important immune tissue hemocyte, gill, and epithelium, and high in muscle and heart with levels 11.19-fold and 13.29-fold over that in hepatopancreas, respectively (Fig. 5A).

### Expression of LvCactus in hemocytes of pathogen- and stimulant-challenged shrimp

The shrimp hemocytes are leukocyte-like blood cells with phagocytic functions, which involve in immune defense and construct an important immune tissue. The expression of LvCactus in hemocytes from *L. vannamei* during LPS, poly (I:C), *V. parahemolyticus*, *S. aureus*, and WSSV challenges were detected using real-time RT-PCR with expression level at 0 h as the baseline. In response to LPS, LvCactu showed a periodic expression profile with 2 peaks, a 7.71-fold increase at 4 hpi and a 13.66-fold increase at 24 hpi, followed by a sharp decrease after 24 hpi (Fig. 5B). In the *V. parahemolyticus* challenged *L. vannamei* hemocytes, LvCactus was dramatically up-regulated during the first 8 hpi and reached a peak of 12.91-fold relative to the baseline, and then sharply decreased to low levels at 12–48 hpi, and finally increased again at 72 hpi with a 5.76-fold level over the baseline (Fig. 5C). During the *S. aureus* infection, the LvCactus expression maintained high levels after 0 hpi with a peak at 24 hpi (7.32-fold increase) (Fig. 5D). Compared with the former challenge groups, the response of LvCactus expression to poly (I:C) is moderate with a highest level of 3.12-fold increase at 48 hpi (Fig. 5E). Unlike the stimulant and bacterial challenge groups, in hemocytes from WSSV-infected *L. vannamei*, the LvCactus expression levels kept decreasing after 0 hpi and reached rock-bottoma rock-bottom level of 62.8% decrease at 48 hpi, and a brief rallyrallied slightly at 72 hpi (Fig. 5F). The control group injected by PBS showed no obvious change of LvCactus expression (data not shown).

## Discussion

In the previous study, the LvDorsal gene in *L. vannamei* has been identified and function-analyzed [19]. Similar to *D. melanogaster* Dorsal, LvDorsal can translocate into the nucleus and bind the NF-κB motif in promoter regions of multiple immune-associated genes to activate their expressions. In addition, several other immune proteins in Toll/NF-κB pathway of *L. vannamei*, such as Lvtoll1, Lvtoll2, Lvtoll3, and Lvpelle, have also been identified and functional characterized. These proteins have a positive effect on the expression of AMPs, which are secreted to extracellular space to defend against microorganism invaders [19]–[21]. The D. melanogaster Cactus gene, homologous to IκB in vertebrates, has been identified, which can bind Dorsal to inhibit its nuclear translocation and activation functions for immune responses. Responding to extracellular stimuli and intracellular signaling cascades, the Cactus-Dorsal complex can be dissociated by proteasomal degradation of Cactus, thereby releasing Dorsal to translocate into the nucleus [40]–[42]. The LvCactus studied here is the first Cactus gene cloned in shrimp, and could be an important regulating factor in immune system of *L. vannamei*. LvCactus showed sequence similarities to other Cactus proteins from arthropods, and could be clustered into the insect group by full-length analysis using NJ method, suggesting they could have similar functions.

In mammalian IκB, the IκB degradation motif is the IκB kinase (IKK) targeting site, in which the conserved serine residues can be phosphorylated in response to extracellular stimuli leading to ubiquitination and proteasomal degradation of IκB [43], [44]. LvCactus also contains a conserved IκB degradation motif (DS_57_GFLS_61_), suggesting LvCactus may be similarly regulated by IKK activation. The ANK domains, about 33 amino acids long and normally occurring in at least four consecutive copies, generally serve as sites of protein-protein interaction [45]. In IκB, the ANK domains bind with the Rel homology domain of NF-κB and mask the nuclear localization signal (NLS) to prevent NF-κB nuclear translocation [46]. Architecture analysis shows that LvCactus has six ANK domains, suggesting a possible interaction between LvCactus and the NF-κB homologue in *L. vannamei*. LvCactus also contains a PEST sequence, which is normally present in IκB to regulate the degradation of free IκB and can be masked by the NF-κB interaction [47]. In free IκB, the PEST sequence is exposed and can be phosphorylated, leading to the degradation of free IκB [48], [49]. Unlike in most IκB protein from mammals, the PEST sequence in LvCactus is predicted to localize in the N-terminus region but not in the C-terminus, indicating a different structure of LvCactus with mammalian IκB.

Dual-luciferase reporter assays showed that contrary to that of LvDorsal, over-expression of LvCactus can down-regulate the activities of shrimp AMPs promoters, suggesting that LvCactus could be an inhibitor of LvDorsal. In *Drosophila*, interactions between Cactus and NF-κB factor Dorsal have been reported [39]. In our study, immunoprecipitation assays showed that LvCactus interacts with LvDorsal, and fluorescence microscopy demonstrated that in LvCactus over-expressing cells LvDorsal was retained in cytoplasm even after LPS stimulation, suggesting LvCactus could inhibit the function of LvDorsal in immune response through binding LvDorsal and preventing its nuclear translocation.

We found that the expression level of LvCactus was increased after LPS, poly(I:C), *V. parahemolyticus*, and *S. aureus* injection and reached peaks at 4–8 hpi, suggesting a rapid activation response of LvCactus to bacteria and immune stimulants challenges. Numerous studies have reported that the NF-κB pathway of shrimp is activated during bacterial infection and immune stimulant challenge [17], [19], [20], [50], [51]. The promoter of LvCactus contains five putative NF-κB binding motifs, among which four have been proved by ChIP to be bound by LvDorsal. By dual-luciferase reporter assays, we also observed that the transcriptional activity of LvCactus promoter can be increased by LvDorsal expression. So, the up-regulation of LvCactus expression during bacteria and immune stimulants challenges could be caused by NF-κB pathway activation, and may work as a negative feedback to regulate NF-κB pathway functions to in turn inhibit the translocation of LvDorsal leading to the decrease of LvCactus expression and the relief of the inhibition of LvDorsal translocation. This process repeated and made the expression of LvCactus showing periodic fluctuations during bacteria and stimulants challenges, particularly in the LPS-stimulated group, as shown in Fig. 5. The mechanism for the self-inhibitory regulation of LvCactus expression shown by dual-luciferase reporter assays could also function through the LvCactus/LvDorsal feedback regulatory pathway. These indicate a tight regulation network between LvCactus and LvDorsal (Fig. 6).

In view that previous studies have shown that WSSV challenge can also activate the Toll/NF-κB pathway [20], [51], it would be predicted that LvCactus expression could be enhanced by LvDorsal activation during WSSV infection. However, real-time RT-PCR showed that unlike bacteria and immune stimulants challenges, the LvCactus expression level persistently decreased throughout the course of WSSV infection with a bottom at 48 hpi with a value only 37.2% of that before challenge, maybe indicating that LvCactus performs different functions in WSSV and bacterial infections. Moreover, it has been reported that LvDorsal can enhance the expression of several WSSV viral immediate-early genes, such as wsv069 (ie1), wsv303 and wsv371, by binding to the NF-κB binding motifs in their promoter regions [20], [51]. On the other hand, a WSSV viral immediate-early gene WSSV499 can also activate the Toll-mediated NF-κB pathway to indirectly promote expressions of other viral genes [20]. Our study here indicates an unknown mechanism adopted by WSSV, which could break the LvCactus and LvDorsal feedback regulatory network and further activate the NF-κB pathway to promote expression of many viral genes and facilitate virus proliferation cycles. The relation between LvCactus functions and WSSV infection is worthy of further studies.

## Supporting Information

Figure S1 **Nucleotide and deduced amino acid sequences of LvCactus.** The ORF of the nucleotide sequence is shown in upper-case letters, while the promoter, 5′ and 3′-UTRs sequences are shown in lowercase. Nucleotides and amino acids are numbered on the left of the sequences. The IκB binding motifs in the promoter region are shown with blue letters, while the transcription start site is shaded in red, and the poly A signal is boxed. Amino acid sequence is represented with one-letter codes above the nucleotide sequence. The ankyrin repeats are shown with red letters, while the PEST sequence is underlined with black line, and the IκB degradation motif is underlined with red line, on which the two conserved serine residues are shaded in green.
(TIF)

Figure S2 **Subcellular localization of LvCactus-GFP fusion protein in S2 cell.** Drosophila S2 cells were transfected with pAc5.1-LvCactus-GFP, treated with Hochest 33258 to counterstain nuclei (blue), and observed with confocal laser scanning microscope. The LvCactus-GFP fusion protein (green) was detected within the cytoplasm.
(TIF)
