# Supplementary figures and images for: Identification, Characterization, and Function Analysis of the Cactus Gene from Litopenaeus vannamei
Source: PLoS One. 2012 Nov 21;7(11):e49711. doi: 10.1371/journal.pone.0049711 (PMC3504109; doi:10.1371/journal.pone.0049711)

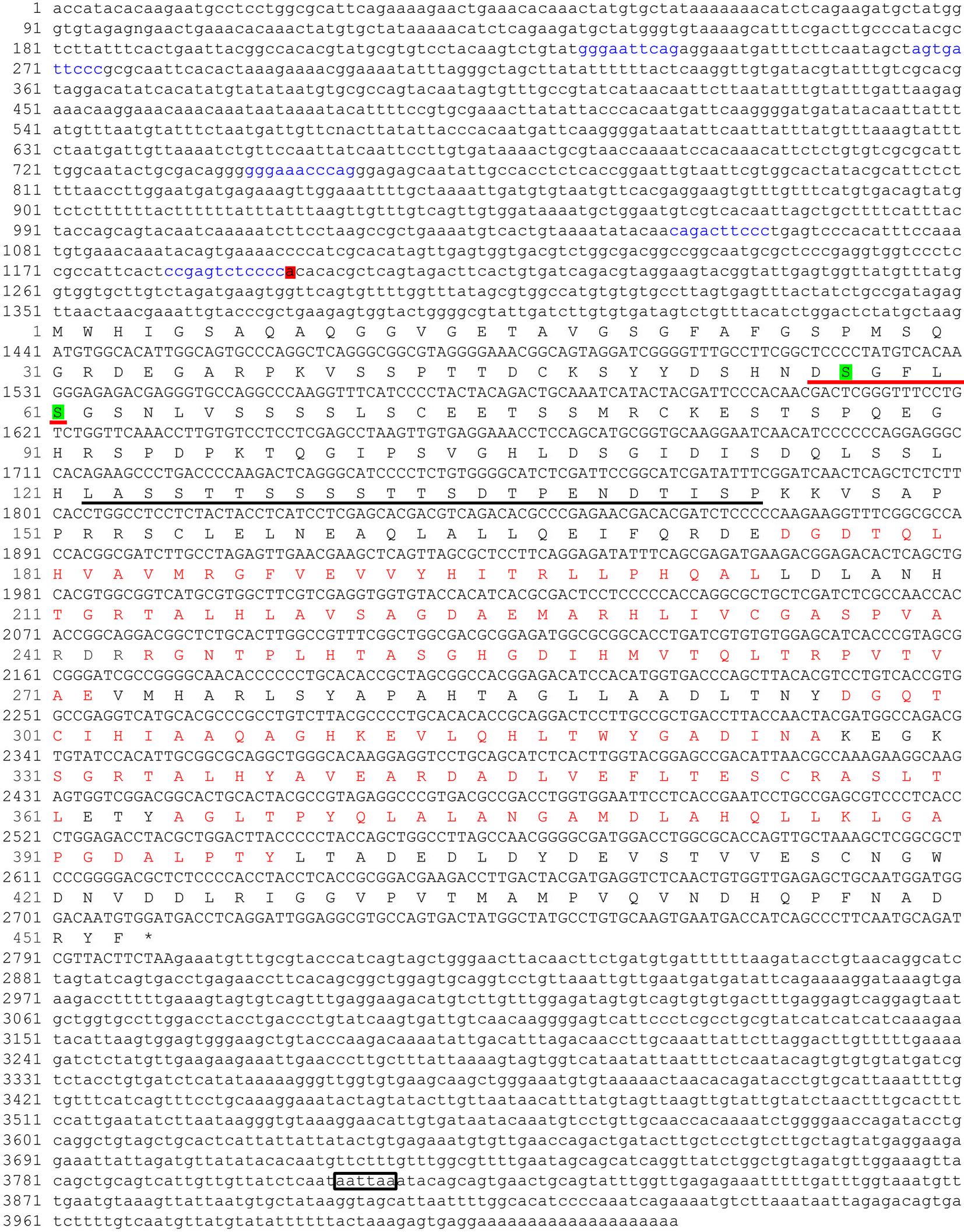

Supplement: Figure S1 — Nucleotide and deduced amino acid sequences of LvCactus. The ORF of the nucleotide sequence is shown in upper-case letters, while the promoter, 5′ and 3′-UTRs sequences are shown in lowercase. Nucleotides and amino acids are numbered on the left of the sequences. The IκB binding motifs in the promoter region are shown with blue letters, while the transcription start site is shaded in red, and the poly A signal is boxed. Amino acid sequence is represented with one-letter codes above the nucleotide sequence. The ankyrin repeats are shown with red letters, while the PEST sequence is underlined with black line, and the IκB degradation motif is underlined with red line, on which the two conserved serine residues are shaded in green. (TIF) [file pone.0049711.s001.tif]

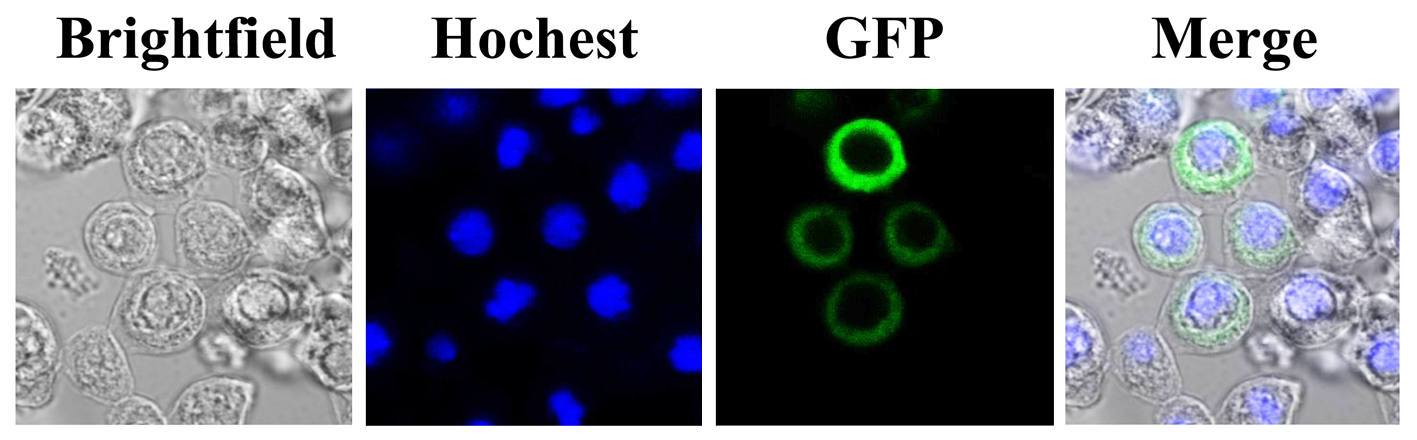

Supplement: Figure S2 — Subcellular localization of LvCactus-GFP fusion protein in S2 cell. Drosophila S2 cells were transfected with pAc5.1-LvCactus-GFP, treated with Hochest 33258 to counterstain nuclei (blue), and observed with confocal laser scanning microscope. The LvCactus-GFP fusion protein (green) was detected within the cytoplasm. (TIF) [file pone.0049711.s002.tif]
